# The adjusted ferritin inflammation index: a novel metric for predicting mortality in heart failure with reduced and mildly reduced ejection fraction

**DOI:** 10.1093/eschf/xvag028

**Published:** 2026-01-21

**Authors:** Çetin Alak, Şükrü Çiriş, Furkan Fatih Yurdalan, Fazil Çağrı Hunutlu, Zeynep Kumral, Tunay Şentürk

**Affiliations:** Department of Cardiology, Faculty of Medicine, Bursa Uludag University, Bursa, Turkey; Department of Cardiology, Gaziantep State Hospital, Gaziantep, Turkey; Department of Cardiology, Faculty of Medicine, Bursa Uludag University, Bursa, Turkey; Division of Hematology, Department of Internal Medicine, Faculty of Medicine, Bursa Uludag University, Bursa, Turkey; Department of Cardiology, Unye State Hospital, Ordu, Turkey; Department of Cardiology, Faculty of Medicine, Bursa Uludag University, Bursa, Turkey

**Keywords:** Heart failure, Ferritin, Inflammation, Mortality, Biomarkers, AFII (Adjusted Ferritin Inflammation Index)

## Abstract

**Introduction:**

Iron deficiency is a prevalent comorbidity in patients with heart failure (HF) and is associated with adverse outcomes. Traditional markers such as ferritin and transferrin saturation may be misleading due to the confounding impact of systemic inflammation. This study aimed to develop and validate the Adjusted Ferritin Inflammation Index (AFII), a novel composite score integrating ferritin/C-reactive protein (CRP) ratio and albumin levels, to improve mortality risk stratification in HF patients.

**Methods:**

This retrospective cohort study included 322 patients with HF and reduced or mildly reduced ejection fraction (HF with reduced ejection fraction: left ventricular ejection fraction ≤40%; HF with mildly reduced ejection fraction: left ventricular ejection fraction 41%–49%). Patients were evaluated for iron parameters between January 2017 and September 2023. Laboratory values (ferritin, CRP, and albumin) were obtained at admission for inpatients or at the first outpatient evaluation. Baseline characteristics were compared between survivors and deceased patients. Adjusted Ferritin Inflammation Index was derived using logistic regression and calculated as: AFII = (Albumin × −0.168) + (Ferritin/CRP × −0.012) + 6.958. The score was log-transformed (Base 2), and the optimal cut-off (2.1) was determined via receiver-operating characteristic curve analysis. Mortality predictors were assessed using Cox regression, and survival differences were analysed with Kaplan–Meier curves.

**Results:**

During a median follow-up of 41 months, 106 patients (32.9%) died. In multivariate Cox regression, AFII ≥ 2.1 independently predicted mortality (hazard ratio: 2.155; 95% confidence interval: 1.361–3.412; *P* = .001), along with New York Heart Association (NYHA) class, sodium, brain natriuretic peptide, and smoking. Ferritin and transferrin saturation were not associated with survival (*P* = .733 and *P* = .790, respectively). The AFII showed superior predictive performance [area under the curve (AUC): 0.713] compared with ferritin/CRP (AUC: 0.438) and albumin (AUC: 0.694). Kaplan–Meier analysis showed significantly reduced survival in patients with AFII ≥ 2.1 across the overall cohort (3-year survival: 54.9% vs 84.6%).

**Conclusion:**

Adjusted Ferritin Inflammation Index is a novel inflammation-adjusted metric that independently predicts mortality in HF with reduced ejection fraction/HF with mildly reduced ejection fraction patients and outperforms traditional iron markers. Its use may enhance risk stratification and inform future strategies for iron deficiency management in HF.

## Graphical Abstract

**Graphical Abstract:**
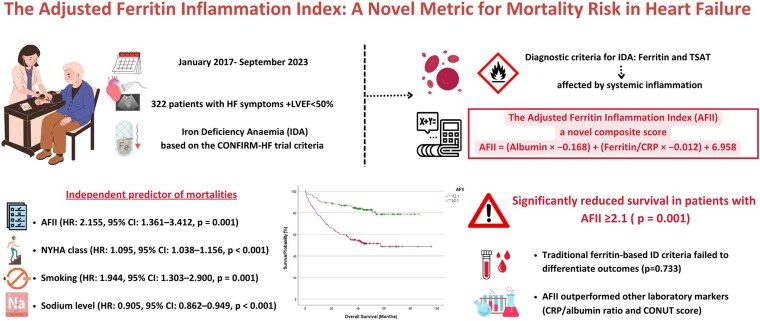
Systemic inflammation alters ferritin levels and limits the prognostic utility of conventional iron-deficiency markers in heart failure. The Adjusted Ferritin Inflammation Index (AFII), integrating serum albumin and the ferritin/C-reactive protein ratio, was developed to account for this inflammatory confounding. In a cohort of 322 patients with heart failure with reduced ejection fraction/heart failure with mildly reduced ejection fraction, AFII independently predicted all-cause mortality, whereas traditional ferritin-based criteria were not prognostic. An AFII ≥2.1 identified patients with significantly reduced survival.

## Introduction

Iron deficiency (ID) is a pervasive and clinically significant condition in individuals with heart failure (HF), affecting ∼50% of subjects with chronic HF. This comorbidity has been consistently linked to poorer functional ability, degraded quality of life, and elevated hospitalization rates.^1^ Importantly, HF itself is increasingly recognized as a chronic inflammatory state, characterized by sustained elevations in inflammatory cytokines such as interleukin-6 (IL-6), interleukin-1β, and tumour necrosis factor-α (TNF-α), as well as increased levels of C-reactive protein (CRP) and high-sensitivity CRP (hs-CRP).^2^ This systemic inflammation not only contributes to disease progression but also interferes with diagnostic markers of ID. Ferritin, as an acute-phase reactant, may be elevated in the presence of inflammation, obscuring true iron status.

Current international guidelines recommend intravenous (i.v.) iron therapy for symptomatic HF subjects with HF with reduced ejection fraction (HFrEF)/HF with mildly reduced ejection fraction (HFmrEF) and ID, described by serum ferritin <100 or 100–299 ng/ml with transferrin saturation (TSAT) <20%. These recommendations are supported by robust evidence from randomized controlled trials, such as FAIR-HF and CONFIRM-HF, demonstrating improved exercise capacity and reduced HF-related hospitalizations. However, a consistent reduction in mortality has not been observed, highlighting the limitations of current ID diagnostic criteria and therapeutic strategies.^3–5^

One major challenge in accurately assessing ID in HF patients is inflammation's confounding role. Ferritin, an acute-phase reactant, is elevated during systemic inflammation, potentially obscuring the detection of true ID. This is particularly relevant in HF, a chronic condition characterized by low-grade systemic inflammation. The reliance on ferritin and TSAT as markers of iron status may fail to differentiate between inflammation-driven elevations in ferritin and actual iron stores, potentially leading to misclassification and suboptimal treatment strategies. Recent studies underscore the need for innovative approaches to disentangle ferritin’s dual role as a marker of both HF iron storage and inflammation.^6,7^

To overcome these limitations, we propose an inventive composite index, the Adjusted Ferritin Inflammation Index (AFII), incorporating the ferritin/CRP ratio and albumin levels. This index aims to account for the confounding effects of inflammation on ferritin levels, providing a more precise assessment of iron status in HF patients. By integrating inflammation and nutritional status biomarkers, AFII offers a comprehensive approach to evaluating the interplay between ID, inflammation, and clinical outcomes in HF.

This study proposes a novel approach to assess ID by introducing the AFII, a composite score that incorporates the ferritin/CRP ratio and albumin levels. This index aims to correct for the confounding effects of inflammation on ferritin measurements, thereby providing a more dependable assessment of iron status in HF patients. Using data from a well-characterized cohort, we tested the prognostic worth of AFII in predicting mortality and compared its performance with existing diagnostic parameters. Adjusted Ferritin Inflammation Index is not intended to replace established clinical criteria but rather to serve as a complementary prognostic tool. By addressing the limitations of conventional markers and integrating indicators of inflammation and nutrition, AFII may refine risk stratification, enhance prognostic accuracy, and support more targeted therapeutic strategies in high-risk HF patients.

## Methods

### Study design and population

This retrospective cohort study intended to measure the predictive worth of AFII in HF cases. The data were sourced from 1968 patients who presented with HF symptoms and an EF below 50% and were evaluated at a single tertiary care centre among 1 January 2017 and 1 September 2023. Patients were classified as HFrEF (≤40%) or HFmrEF (41%–49%). Patients were screened based on the inclusion and exclusion standards of the CONFIRM-HF trial, which assesses ID and HF outcomes.^8^ Inclusion criteria: age ≥18, left ventricular ejection fraction (LVEF) <50%, availability of ferritin/CRP/albumin, and complete follow-up data. Exclusion criteria: absence of detailed iron parameter assessments at admission, active infection, malignancy, dialysis or end-stage renal disease, recent surgery, valve replacement, coronary angiography within last 3 months, and haemoglobin >16 g/dl (men) or >15 g/dl (women).

**Figure 1:**
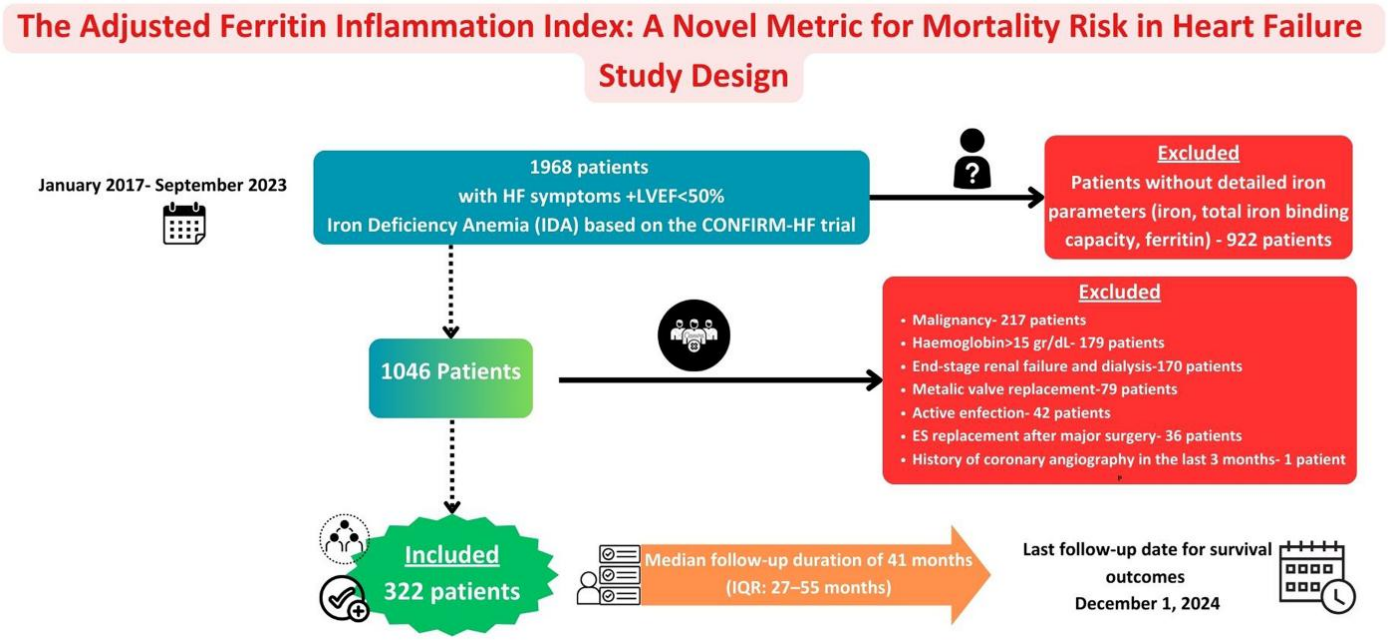
Study design and patient selection for the assessment of the Adjusted Ferritin Inflammation Index as a mortality risk metric in heart failure

A total of 1646 patients were eliminated, leaving 322 patients for ultimate assessment. The last follow-up date for survival outcomes was 1 December 2024 (*Figure 1*). Mortality data were obtained from the national death registry and cross-validated with hospital electronic medical records and follow-up documentation.

### Data collection and Adjusted Ferritin Inflammation Index score development

Patients were either hospitalized for acute decompensated HF or evaluated in the outpatient clinic for stable HF. Baseline demographic, clinical, and laboratory data were collected, including ferritin, CRP albumin, haemoglobin, TSAT, total cholesterol, HDL, LDL, and lymphocyte count. Nutritional status was assessed by the CONUT score. Laboratory parameters were obtained at admission (inpatients) or at the index visit (outpatients).

To derive the AFII score, logistic regression analysis was used for score construction only, not for outcome prediction. Initially, inflammation-related biochemical markers (ferritin/CRP ratio, CRP/albumin ratio, and CONUT score) were evaluated using univariate Cox regression. Subsequently, binary logistic regression with the forward likelihood ratio method was used to identify the optimal combination of variables for score derivation. The final equation included ferritin/CRP and albumin, which improved model performance without collinearity. High-density lipoprotein slightly improved the area under the curve (AUC: 0.724) but was excluded due to marginal gain. Thus, the final model was adopted, incorporating only ferritin/CRP ratio and albumin (AUC: 0.713). The AFII score was derived using logistic regression coefficients as follows:


AFII=(Albumin×−0.168)+(Ferritin/CRP×−0.012)+6.958.

The score was log-transformed (Base 2), and an optimal cut-off point of 2.1 was determined via receiver-operating characteristic (ROC) curve analysis using the Youden index. External validation emphasized as essential for future research.

### Statistical analysis

Data were analysed using SPSS version 28.0 (IBM, NY, USA). Continuous variables were represented as the average ± standard deviation or median with interquartile range (IQR), depending on the distribution, and categorical variables as counts and percentages. Normality was evaluated using the Kolmogorov–Smirnov test. For comparisons between groups, Student’s *t*-test was employed for normally distributed continuous variables, while Mann–Whitney *U* test was used for non-normally distributed variables.

The predictive ability of AFII, ferritin/CRP, and albumin for mortality was evaluated using ROC curve analysis. The AUC and 95% confidence intervals (CIs) were calculated. Kaplan–Meier survival curves were generated and compared using the log-rank test.

Cox proportional hazards regression analysis was used to identify independent predictors of mortality:

1. Univariate analysis was performed to screen candidate variables (*P* < .20),
2. Multivariate analysis identified independent predictors.

Hazard ratios (HRs) were derived exclusively from Cox models. Odds ratios were applied only during logistic regression used for AFII score construction. Multicollinearity was tested using variance inflation factors (VIFs), with all variables remaining below an acceptable threshold (VIF < 5).

### Validation of the score

The AFII score’s prognostic validity was confirmed through several approaches:

- In multivariate Cox regression, AFII remained an independent predictor of mortality (HR: 2.155, 95% CI: 1.361–3.412, *P* = .001).
- Internal consistency was supported by bootstrap analysis using 1000 resamples (*B* = 1.457, standard error (SE) = 0.273, 95% CI: 0.955–2.040, *P* < .001).
- ROC analysis showed limited performance of ferritin/CRP (AUC = 0.438, *P* = .062) and moderate performance of albumin (AUC = 0.694, *P* < .001), whereas AFII showed superior discrimination (AUC = 0.713).
- To assess external stability, split-sample validation was performed by randomly assigning cases into training (70%) and test (30%) sets using the SPSS function random variable. UNIFORM(0,1), which generates random values from a uniform distribution between 0 and 1. Adjusted Ferritin Inflammation Index retained its discriminatory capacity in both the training set (AUC = 0.668) and test set (AUC = 0.675).
- No significant multicollinearity was found between AFII and its components. Variance inflation factor values were calculated for ferritin/CRP (1.207), albumin (2.513), and AFII (2.738), all remaining well below the commonly accepted threshold of 5.

## Results

### Baseline characteristics and univariate analysis

A total of 322 patients (mean age 64 ± 11 years, 38% female) were included in the trial, with a median follow-up period of 41 months (IQR: 27–55 months). One hundred and six patients (32.9%) died during follow-up, while 216 (67.1%) survived.

Baseline characteristics were compared between the deceased and surviving individuals and are presented in *Table 1*. Patients in the deceased group had significantly higher New York Heart Association (NYHA) class (*P* < .001), CRP levels [5.35 mg/dl (IQR: 1.5–8.45) vs 3.65 mg/dl (IQR: 1–6.2), *P* = .002], CONUT scores [2 (IQR: 1–4) vs 2 (IQR: 1–3), *P* < .001], CRP/albumin ratio [0.094 (IQR: 0.087–0.1) vs 0.141 (IQR: 0.132–0.15), *P* < .001], and AFII [2.24 (IQR: 2.08–2.34) vs 2 (IQR: 1.85–2.16), *P* < .001].

**Table 1:** Baseline characteristics of deceased and surviving heart failure patients

	Deceased (*n* = 106)	Survived (*n* = 216)	*P*-value
Age, years, median (IQR 1–3)	67.5 (59.5–75.5)	67 (59–75)	.122
Gender, *n* (%)			.229
Female	33 (31.1)	82 (38)	
Male	73 (68.9)	134 (62)	
Hypertension, *n* (%)	85 (80.2)	165 (76.4)	.442
Diabetes mellitus, *n* (%)	61 (57.5)	101 (46.8)	.069
Coronary artery heart disease, *n* (%)	84 (79.2)	135 (62.5)	**.007**
Active smoking, *n* (%)	63 (59.4)	97 (44.9)	**.014**
Ejection fraction, median (IQR)	30 (22–35)	38 (30–45)	**<.001**
Intravenous iron therapy, *n* (%)	14 (13.2)	28 (13)	.951
ACE inhibitors/ARBs/ARNi, *n* (%)	76 (71.7)	174 (80.6)	.073
Beta-blockers, *n* (%)	97 (91.5)	190 (88)	.337
Mineralocorticoid receptor antagonists, *n* (%)	56 (52.8)	111 (51.4)	.808
NYHA classification, *n* (%)			**<.001**
I	1 (0.9)	64 (29.6)	
II	22 (20.8)	90 (41.7)	
III	51 (48.1)	50 (23.1)	
IV	31 (29.2)	12 (5.6)	
Sodium, mmol/l, median (IQR 1–3)	137 (134–140)	138 (136–140)	**<.001**
Potassium, mmol/l, median (IQR 1–3)	4.4 ± 0.6	4.3 ± 0.5	.258
Chloride, mmol/l, median (IQR 1–3)	102.5 (99–105)	104 (101–106)	**.007**
Haemoglobin, g/dl, median (IQR 1–3)	11.7 (10.5− 13)	12.4 (11.1–13.2)	**.021**
White blood cells, 10^9^/l, median (IQR 1–3)	8.0 ± 2.0	8.2 ± 1.9	.453
Lymphocytes, 10^9^/l	1500 (1.045–1.975)	1755 (1.285–2.265)	**<.001**
Iron, μg/dl	40 (30.25–70.25)	49 (34–83)	**.003**
Ferritin, ng/dl	52.5 (30–108.25)	59 (30–100.5)	.733
Transferrin saturation group			
Group 1 <%20			
Group 2 ≥%20			
Ferritin group, ng/dl, *n* (%)			.790
Group 1 <100	76 (71.7)	163 (75.5)	
Group 2 Transferrin saturation <%20 and 100–299	15 (14.2)	26 (12)	
Group 3 Transferrin saturation >%20 and 100–299	10 (9.4)	15 (6.9)	
Group 4 ≥300	5 (4.7)	12 (5.6)	
Total cholesterol, mg/dl	151 (128–194)	167.5 (14.5–205.5)	.089
LDL, mg/dl	91.5 (77–123.5)	99.5 (73–125.75)	.341
HDL, mg/dl	36 (29.25–45.5)	42 (33.75–50.5)	**<.001**
Triglyceride, mg/dl	112.5 (72.5–146.75)	112 (80–160.75)	.591
Creatinine, mg/dl	1.2 (1.05–1.67)	1.03 (0.88–1.3)	**<.001**
eGFR, ml/min/1.73 m^2^	59 (42.5–80)	70 (52.5–87.5)	**.002**
CRP, mg/dl	5.35 (1.5–8.45)	3.65 (1–6.2)	**.002**
Albumin, g/l	38 (36–40)	41 (39–44)	**<.001**
CONUT score	2 (1–4)	2 (1–3)	**<.001**
CRP/albumin ratio	0.094 (0.087–0.1)	0.141 (0.132–0.15)	**<.001**
Ferritin/CRP ratio	11.1 (7.8–14.1)	13.5 (8–14.5)	.071
Adjusted Ferritin Inflammation Index	2.24 (2.08–2.34)	2 (1.85–2.16)	**<.001**
First admission			**<.001**
Outpatient, *n* (%)	52 (49.1)	55 (25.5)	
Inpatient, *n* (%)	54 (50.9)	161 (74.5)	

Notes: Bold values indicate statistically significant differences between deceased and surviving patients (*P* < .05). ACE inhibitors, angiotensin-converting enzyme inhibitors; ARBs, angiotensin receptor blockers; ARNi, angiotensin receptor–neprilysin inhibitor; CONUT score, controlling nutritional status score; CRP, C-reactive protein; eGFR, estimated glomerular filtration rate; IQR, interquartile range; NYHA, New York Heart Association classification.

Conversely, no notable disparities were detected between the deceased and surviving groups regarding age [67.5 years (IQR: 59.5–75.5) vs 67 years (IQR: 59–75), *P* = .122], ferritin levels [52.5 ng/ml (IQR: 30–108.25) vs 59 ng/ml (IQR: 30–100.5), *P* = .733], triglyceride levels [112.5 mg/dl (IQR: 72.5–146.75) vs 112 mg/dl (IQR: 80–160.75), *P* = .591], and TSAT groups (*P* = .284), indicating that neither ferritin nor TSAT levels were significant determinants of survival in univariate analysis (*Table 1*).

### Baseline characteristics and univariate analysis according to Adjusted Ferritin Inflammation Index

Adjusted Ferritin Inflammation Index ≥2.1 was identified as the optimal cut-off (Youden index). Patients with AFII ≥2.1 had significantly higher age (69 vs 65 years, *P* = .005), and notably, a worse LVEF (32% vs 38%, *P* = .005) compared with those with AFII < 2.1. Furthermore, subjects in the higher AFII group exhibited a greater prevalence of coronary artery disease (74.2% vs 61.6%, *P* = .015) and CRP levels were significantly increased in subjects with AFII ≥ 2.1 (5.5 vs 3.07 mg/dl, *P* < .001; *Table 2*).

**Table 2:** Baseline characteristics and univariate analysis according to Adjusted Ferritin Inflammation Index (AFII)

	AFII < 2.1 (*n* = 159)	AFII ≥ 2.1 (*n* = 163)	*P*-value
Age, years, median (IQR 1–3)	65 (56–71)	69 (61–76)	.**005**
Gender			.678
Female, *n* (%)	55 (47.8)	60 (52.2)	
Male, *n* (%)	104 (50.2)	103 (49.8)	
Hypertension, *n* (%)	122 (76.7)	128 (78.5)	.699
Diabetes mellitus, *n* (%)	77 (48.4)	85 (52.1)	.505
Coronary artery heart disease, *n* (%)	98 (61.6)	121 (74.2)	.**015**
Active smoking, *n* (%)	80 (50.3)	80 (49.1)	.**825**
Ejection fraction, median (IQR)	38 (30–45)	32 (25–40)	.**005**
ACE inhibitors/ARBs/ARNi, *n* (%)	127 (79.9)	123 (75.5)	.342
Beta-blockers, *n* (%)	134 (84.3)	153 (93.9)	.**006**
Mineralocorticoid receptor antagonists, *n* (%)	78 (49.1)	89 (54.6)	.319
NYHA classification, *n* (%)			**<**.**001**
I	49 (30.8)	16 (9.8)	
II	58 (36.5)	55 (33.7)	
III	40 (25.1)	61 (37.4)	
IV	12 (7.5)	31 (19)	
Sodium, mmol/l, median (IQR 1–3)	138 (136–140)	137 (135–140)	.**096**
Potassium, mmol/l, median (IQR 1–3)	4.3 (4–4.67)	4.4 (4–4.72)	.190
Chloride, mmol/l, median (IQR 1–3)	104 (100–108)	103 (99–108)	.**213**
Haemoglobin, g/dl, median (IQR 1–3)	12.8 (11.9–13.9)	11.7 (10.6–12.8)	**<**.**001**
White blood cells, 10^9^/l, median (IQR 1–3)	8.23 ± 1.90	8.08 ± 2.02	.501
Lymphocytes, 10^9^/l	1.96 (1.39–2.42)	1.48 (1.00–2.01)	**<**.**001**
Iron, μg/dl	59 (28–90)	38 (23–50)	**<**.**001**
Ferritin, ng/dl	60 (20–100)	47 (19–80)	.076
Transferrin saturation group			.**015**
Group 1 <%20	99 (62.3)	122 (74.8)	
Group 2 ≥%20	60 (37.7)	41 (25.2)	
Ferritin group, ng/dl, *n* (%)			.140
Group 1 <100	114 (71.7)	125 (76.7)	
Group 2 Transferrin saturation <%20 and 100–299	19 (11.9)	22 (13.5)	
Group 3 Transferrin saturation >%20 and 100–299	13 (8.2)	12 (7.4)	
Group 4 ≥300	13 (8.2)	4 (2.5)	
Total cholesterol, mg/dl	170 (104–210)	154 (98–195)	.**012**
LDL, mg/dl	102 (76–129)	94 (72–117)	.333
HDL, mg/dl	43 (33–53)	39 (30–46)	**<**.**001**
Triglyceride, mg/dl	125 (106–197)	105 (79–148)	.**004**
Creatinine, mg/dl	1.03 (0.86–1.26)	1.15 (0.95–1.53)	.**002**
eGFR, ml/min/1.73 m^2^	72 (53–87)	61 (45–81)	**<**.**001**
CRP, mg/dl	3.07 (1.9–6.1)	5.5 (2.9–9.9)	**<**.**001**
Albumin, g/l	43 (41–44)	37 (34–38)	**<**.**001**
First admission			.**167**
Outpatient, *n* (%)	112 (70.4)	103 (63.2)	
Inpatient, *n* (%)	47 (29.6)	60 (36.8)	

Notes: Bold values indicate statistically significant differences between patients with AFII < 2.1 and AFII ≥ 2.1 (*P* < .05). AFII, Adjusted Ferritin Inflammation Index; CRP, C-reactive protein; eGFR, estimated glomerular filtration rate; NYHA, New York Heart Association.

These findings underline the association of a higher AFII with poorer clinical characteristics, including more severe coronary artery disease and worse inflammation and nutritional status, which are crucial factors influencing HF prognosis.

### Cox regression analysis

Univariate Cox regression identified several variables associated with mortality (*Table 3*). In multivariate Cox regression, AFII ≥ 2.1 remained an independent predictor of mortality (HR: 2.155, 95% CI: 1.361–3.412, *P* = .001), even after adjusting for NYHA class, sodium, brain natriuretic peptide (BNP), smoking, and estimated glomerular filtration rate. Other independent predictors included NYHA class (HR: 1.095, 95% CI: 1.038–1.156, *P* < .001), sodium (HR: 0.905, 95% CI: 0.862–0.949, *P* < .001), BNP (HR: 1.000, 95% CI: 1.000–1.001, *P* < .001), and smoking (HR: 1.944, 95% CI: 1.303–2.900, *P* = .001). Ferritin and TSAT were not significant predictors of mortality.

**Table 3:** Univariate and multivariate Cox regression analysis of clinical and laboratory parameters

Factor	Univariate analysis				Multivariate analysis			
	HR	%95 CI		*P-*value	HR	%95 CI		*P-*value
		Lower	Upper			Lower	Upper	
Sodium	0.910	0.871	0.952	**<**.**001**	0.905	0.862	0.949	**<**.**001**
Age	1.015	0.999	1.032	.071	1.015	0.999	1.032	.073
Smoking	1.693	1.149	2.496	.**008**	1.944	1.303	2.900	.**001**
NYHA	2.893	2.304	3.631	**<**.**001**	1.095	1.038	1.156	**<**.**001**
BNP	1.000	1.000	1.001	**<**.**001**	1.000	1.000	1.001	<.001
AFII	2.201	1.698	2.852	**<**.**001**	2.155	1.361	3.412	.**001**
HT	1.201	0.744	1.936	.453				
DM	1.423	0.968	2.092	.073				
CAD	1.996	1.248	3.193	.**004**				
EF	0.956	0.936	0.976	**<**.**001**				
Potassium	1.216	0.859	1.722	.269				
Chloride	0.944	0.910	0.980	.**002**				
Haemoglobin	0.848	0.748	0.961	.**010**				
WBC	0.970	0.879	1.070	.542				
Lymphocyte	0.588	0.438	0.788	**<**.**001**				
Iron	0.988	0.980	0.997	.**008**				
Ferritin	1.000	0.998	1.002	.866				
Total cholesterol	0.995	0.991	1.000	.**031**				
HDL	0.971	0.956	0.987	**<**.**001**				
LDL	0.996	0.991	1.001	.141				
Triglycerides	0.999	0.996	1.002	.368				
Creatinine	2.263	1.465	3.495	**<**.**001**				
eGFR	0.987	0.979	0.996	.**004**				
CRP	1.034	1.008	1.061	.**010**				
Albumin	0.874	0.833	0.916	**<**.**001**				
CONUT score	1.285	1.156	1.428	**<**.**001**				

Notes: Bold values indicate statistically significant associations in Cox regression analysis (*P* < .05). AFII, Adjusted Ferritin Inflammation Index; BNP, brain natriuretic peptide; CAD, coronary artery disease; CONUT score, controlling nutritional status score; CI, confidence interval; CRP, C-reactive protein; DM, diabetes mellitus; EF, ejection fraction; eGFR, estimated glomerular filtration rate; HR, hazard ratio; HT, hypertension; Lymphocyte, lymphocyte count; NYHA, New York Heart Association classification; WBC, white blood cells.

### Survival analysis

Kaplan–Meier survival analysis was carried out to examine the survival probabilities across different AFII groups. At various time points, significant differences in survival rates were noted across patients with low and high AFII.

**Figure 2:**
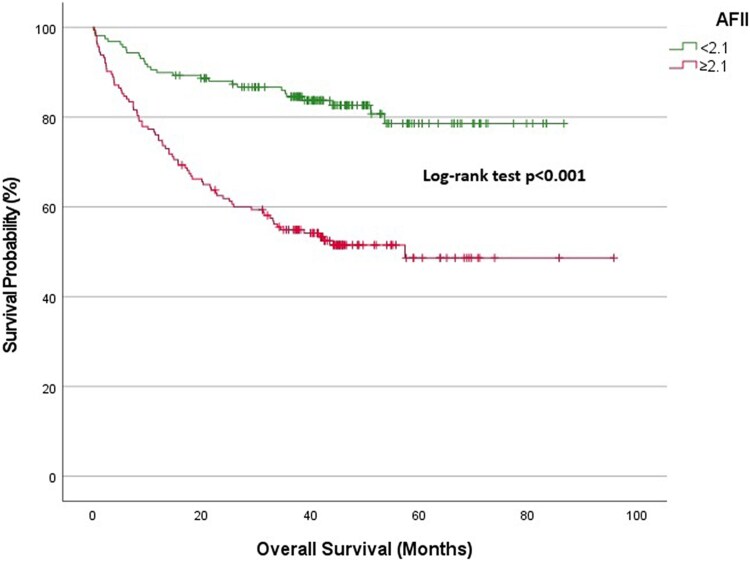
Kaplan–Meier survival analysis for the overall cohort stratified by Adjusted Ferritin Inflammation Index levels (<2.1 vs ≥2.1)

**Figure 3:**
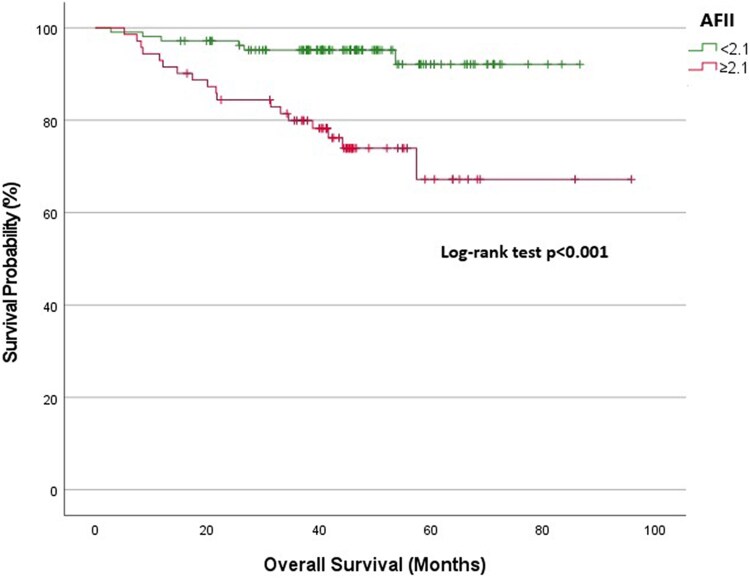
Kaplan–Meier survival analysis for the New York Heart Association Classes I and II patient group stratified by Adjusted Ferritin Inflammation Index levels (<2.1 vs ≥2.1)

**Figure 4:**
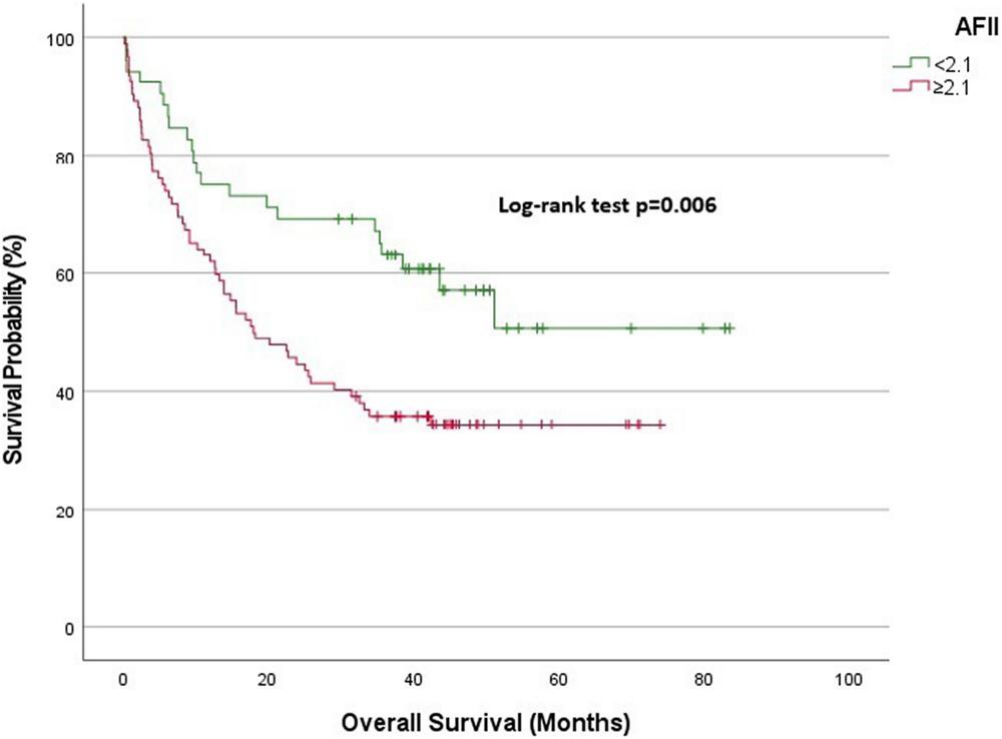
Kaplan–Meier survival analysis for the New York Heart Association Classes III and IV patient group stratified by Adjusted Ferritin Inflammation Index levels (<2.1 vs ≥2.1)

**Figure 5:**
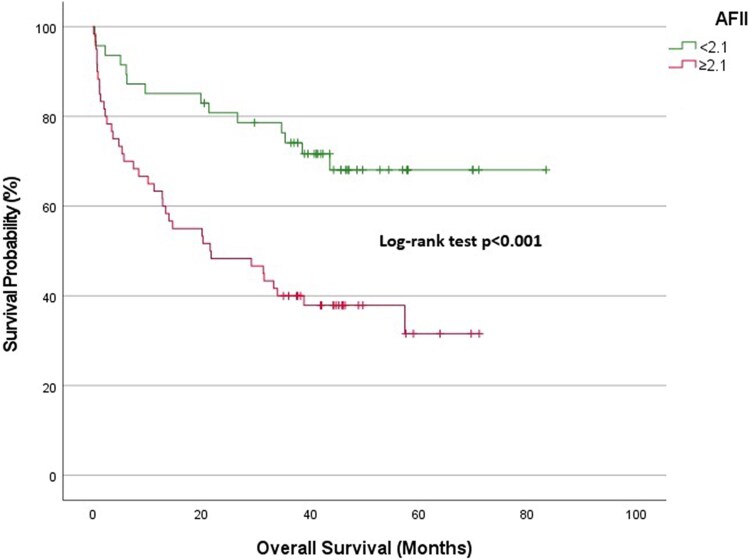
Kaplan–Meier survival analysis for the inpatient patient group stratified by Adjusted Ferritin Inflammation Index levels (<2.1 vs ≥2.1)

**Figure 6:**
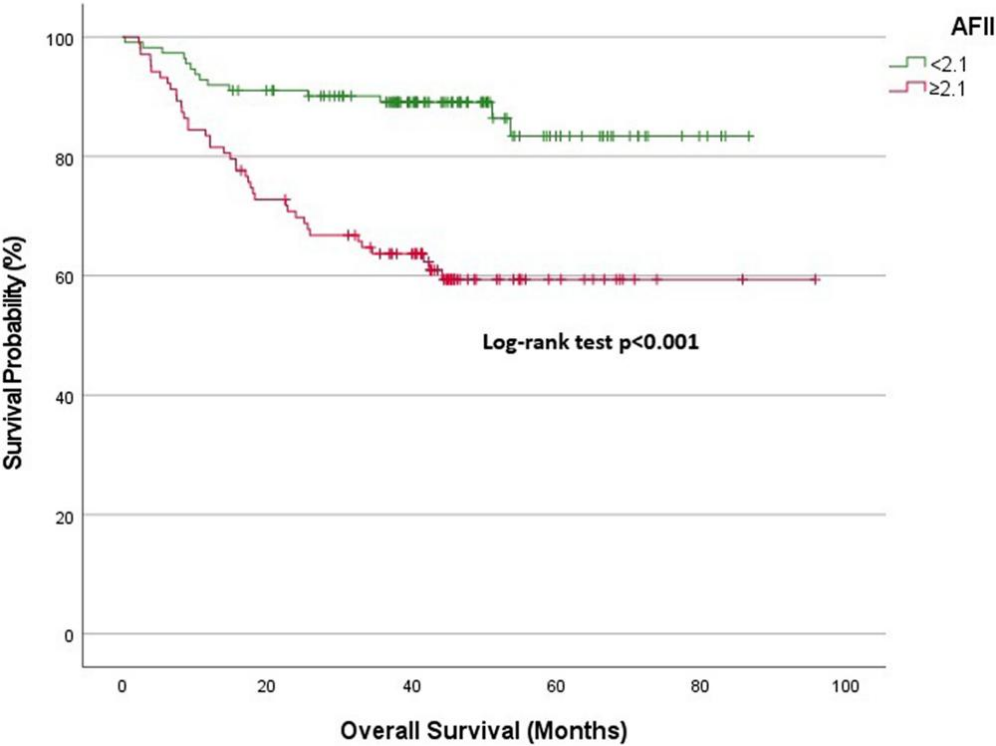
Kaplan–Meier survival analysis for the outpatient patient group stratified by Adjusted Ferritin Inflammation Index levels (<2.1 vs ≥2.1)

- Overall cohort: At 1-year follow-up, the survival rate was 89.9% in the low AFII group and 76.1% in the high AFII group. At 3 years, the survival rates diverged significantly, with the low AFII group demonstrating a survival rate of 84.6%, compared with 54.9% in the high AFII group (*Figure 2*).
- NYHA Classes I and II: The 1-year survival expectancy was 97.2% for the low AFII group and 93% for the high AFII group. At 3 years, the survival rate for the low AFII group remained high at 95.2%, while the high AFII group declined to 79.9% (*Figure 3*).
- NYHA Classes III and IV: At 1-year, the survival expectancy was 75% for the low AFII group and 63% for the high AFII group. At 3 years, the survival rate for the low AFII group was 63.1%, compared with 35.8% for the high AFII group (*Figure 4*).
- Outpatient group: The 1-year survival expectancy was 92% for the low AFII group and 83.5% for the high AFII group. At 3 years, the low AFII group maintained a survival rate of 89.1%, while the high AFII group had a survival rate of 63.7% (*Figure 5*).
- Inpatient group: The 1-year survival expectancy for the low AFII group was 85.1% and 63.3% for the high AFII group. At 3 years, the low AFII group had a survival rate of 74.1%, while the high AFII group had a survival rate of 40% (*Figure 6*).
- HFrEF group (EF ≤ 40%): At 1 year, survival was 87.5% in the low AFII group compared with 66.5% in the high AFII group. At 3 years, survival declined to 80.3% and 42.6%, respectively. The difference between the two groups was statistically significant (log-rank *P* < .001; Supplementary Fig. S1).
- HFmrEF group (EF 41%–49%): At 1 year, survival was 92.5% in the low AFII group compared with 83.1% in the high AFII group. At 3 years, survival rates were 89.4% and 65.7%, respectively. The difference remained statistically significant (log-rank *P* = .002; Supplementary Fig. S2).

The prognostic significance of AFII was consistent across subgroups, including HFrEF vs HFmrEF, NYHA I and II vs III and IV, and inpatient vs outpatient cohorts. These findings underscore the prognostic significance of AFII in predicting long-term survival. Patients in the high AFII group consistently exhibited worse outcomes across all subgroups, emphasizing the need for tailored clinical management based on AFII levels.

### Discrimination and internal validation

The predictive superiority and internal validity of the AFII score were evaluated using multiple statistical approaches. Individually, the components of AFII demonstrated variable predictive power. The ferritin/CRP ratio yielded poor discriminatory capacity (AUC = 0.438, *P* = .062), while albumin alone showed a moderate association with mortality (AUC = 0.694, 95% CI: 0.632–0.755, *P* < .001). In multivariate regression, albumin remained statistically significant (*P* = .026), whereas ferritin/CRP did not (*P* = .249). Importantly, multicollinearity diagnostics revealed acceptable VIF values for ferritin/CRP (1.207), albumin (2.513), and AFII (2.738), indicating no collinearity issues among these predictors. The composite AFII score, combining both markers, demonstrated superior prognostic performance. Its internal consistency was confirmed via bootstrap analysis (*B* = 1.457, SE = 0.273, 95% CI: 0.955–2.040, *P* < .001). Furthermore, split-sample validation revealed stable discriminatory ability in both training (AUC = 0.668) and test (AUC = 0.675) cohorts, underscoring the generalizability and robustness of the AFII score.

## Discussion

In our study, the AFII score, formulated by the ratio of inflammation parameters, was identified as the most significant predictor of mortality in HF patients with LVEF < 50% who were evaluated for detailed iron parameters as determined by multivariate analysis (HR: 2.155, 95% CI: 1.361–3.412, *P* = .001). Similarly, patients with an AFII ≥ 2.1 demonstrated lower survival rates over a 3-year follow-up period (84.6% vs 54.9%).

Currently, in clinical practice, the criteria for defining ID in HF patients (serum ferritin levels <100 ng/ml or ferritin levels between 100 and 299 ng/ml with TSAT <20%) were first introduced in the FAIR-HF study in 2008.^9^ These criteria have since been widely accepted in subsequent studies. However, it is important to note that the FAIR-HF criteria are based on parameters used in the literature for diagnosing ID in subjects with chronic kidney disease. Subsequently, in a study involving 42 HF subjects who received coronary artery bypass grafting, these parameters were compared with bone marrow iron staining results, which are considered the gold standard for diagnosing ID. The findings revealed that the FAIR-HF criteria demonstrated a sensitivity of 82.4%, specificity of 72.0%, a positive predictive value of 66.7%, and a negative predictive value of 85.7%. According to the FAIR-HF criteria, one-third of patients diagnosed with ID were found to have normal bone marrow iron stores.^10^ Furthermore, the FAIR-HF criteria highlighted that ferritin levels were unrelated to mortality.^11^ However, the FAIR-HF criteria do not account for the confounding impact of systemic inflammation on ferritin levels. Ferritin is an acute-phase reactant, and its elevation in the context of inflammation may lead to misclassification of iron status in HF patients. In contrast AFII was designed to overcome this limitation by incorporating the ferritin/CRP ratio and albumin levels, offering a more individualized and inflammation-adjusted measure.

In our cohort, traditional markers such as serum ferritin and TSAT were not independently associated with mortality. These findings are consistent with previous studies questioning the reliability of unadjusted iron markers in the context of inflammation, especially in patients with chronic diseases like HF.

Conversely, the AFII demonstrated a robust association with mortality (*P* < .001). Adjusted Ferritin Inflammation Index predicted mortality in both HFrEF and HFmrEF, in early (NYHA I and II) and advanced stages, and remained valid after excluding patients with advanced CKD. This highlights AFII’s robustness and potential applicability across diverse subgroups. While the FAIR-HF criteria served as the basis for i.v. iron therapy decisions, AFII may help identify higher-risk patients who might benefit from closer clinical follow-up and prioritization for iron status optimization, by distinguishing inflammation-related ferritin elevation from true ID.

These clinical observations were further supported by robust statistical performance of the score. The predictive superiority and internal validity of the AFII score were demonstrated by ROC, Cox regression, bootstrap, and split-sample validation, underscoring its robustness. These findings collectively highlight AFII as a statistically sound and clinically valuable tool for mortality risk stratification in HF patients.

Further interventional studies are warranted to determine whether AFII-guided iron supplementation offers a superior therapeutic benefit compared with conventional approaches. Additionally, future models incorporating other inflammatory biomarkers—such as IL-6, interleukin-1, TNF-α, or hs-CRP—may further enhance prognostic performance.

In our cohort, 13% of patients received i.v. iron therapy. However, AFII scores did not significantly differ between patients who received i.v. iron and those who did not (*P* = .905), and there was no survival difference based on i.v. iron therapy alone. This observation likely reflects the fact that i.v. iron administration was based on conventional FAIR-HF criteria rather than AFII levels. Therefore, AFII was not used in treatment decision-making. While our findings support AFII as a strong prognostic indicator of mortality, we do not claim it currently serves as a therapeutic guide for i.v. iron therapy. Nonetheless, it is conceivable that patients with high AFII—indicating inflammation-adjusted ID—might represent a subgroup warranting closer monitoring and perhaps more targeted iron repletion strategies. These observations may inform the design of future interventional studies that explore AFII-based treatment algorithms.

These considerations gain further relevance when placed in the context of existing evidence on i.v. iron therapy. In a meta-analysis published in February 2024, which included nine randomized controlled trials, i.v. iron therapy in HF patients was shown to significantly reduce the composite risk of hospitalization for HF (HHF) or cardiovascular death by 16%. Additionally, it diminished the composite risk of hospitalization for any reason or all-cause mortality by 8%. However, these outcomes were primarily guided by reductions in HHF and all-cause hospitalizations.^12^ Consequently, uncertainties remain regarding the impact of i.v. iron therapy on mortality outcomes in this patient population. Meta-analyses of i.v. iron therapy have shown benefits in reducing HF hospitalizations, but uncertainties remain about mortality impact. Within this context, AFII may contribute to refining patient selection and improving risk stratification for future iron repletion trials.

In the patient population included in our study, mortality predictors were also evaluated. Among these, BNP emerged as a statistically significant predictor of mortality. Brain natriuretic peptide is released from the heart in reaction to heightened wall stress, heightened sympathetic tone, and vasoconstriction, serving as a compensatory regulatory mechanism. Elevated circulating levels of natriuretic peptides can integrate cardiovascular and haemodynamic stress from multiple sources, making BNP a crucial marker for predicting mortality in this patient group.^13^ In the multivariate Cox regression analysis, BNP was revealed to be a statistically significant indicator of mortality (HR: 1.000, 95% CI: 1.000–1.001, *P* < .001). However, the HR was equal to 1, indicating that while BNP remains significant, its clinical effect on mortality is minimal. This suggests that BNP, although a key marker in HF prognosis, may have a limited independent impact on predicting mortality when considering other covariates.

The NYHA functional classification, a well-established parameter, has long been used to categorize symptoms in HF patients. A 2019 study by Briongos-Figuero et al. involving HF patients with implantable cardioverter defibrillators (ICDs) reported lethality rates of 6.9% in NYHA I patients, 11% in NYHA II patients (HR: 2.2, 95% CI: 1.1–4.9), and 23.9% in NYHA III patients (HR: 5.5, 95% CI: 2.4–12.7). Similarly, in our study, an increase in NYHA class was discovered to correlate with a higher risk of mortality (HR: 1.095, 95% CI: 1.038–1.156, *P* < .001).^14^

Sodium levels, identified as another predictor of mortality, may exert their effects through various mechanisms. Hyponatraemia can lead to volume overload by triggering inappropriate secretion of antidiuretic hormone, which is affiliated with end-organ dysfunction such as renal and hepatic failure and/or HF. Mechanisms as seen in the renin–angiotensin–aldosterone system, excessive sympathetic nervous system stimulation, and the over release of antidiuretic hormone contribute to water retention and an increase in overall blood volume.^15^ Consequently, the resulting fluid imbalance in patients with limited cardiac reserve may help explain the increased mortality observed (HR: 0.905, 95% CI: 0.862–0.949, *P* < .001).

Smoking was identified as an independent risk factor for mortality in HF patients, with an HR of 1.944 (95% CI: 1.303–2.900, *P* = .001). This finding is consistent with previous studies, such as the Cardiovascular Health Study, which reported that ongoing smoking is associated with poor prognosis and increased mortality in HF patients, as it contributes to elevated inflammation and myocardial injury.^16^ Similarly, our results demonstrate that smoking plays a significant role in exacerbating HF outcomes. The chemical constituents of cigarette smoke can enhance atherosclerosis through endothelial damage mediated by their potent oxidative and inflammatory effects. Moreover, studies have shown that nicotine, through carbon monoxide and oxidative stress, can trigger cardiac fibrosis, leading to structural remodelling and cardiac arrhythmias. Through these mechanisms, smoking may cause systolic and diastolic dysfunction, resulting in worsening symptoms and increased mortality in patients with HF.^17^ The insights of our study highlight the importance of addressing smoking cessation as part of HF management strategies to improve patient outcomes.

However, when comparing baseline characteristics by AFII levels, no significant difference in smoking status was observed between the high and low AFII groups (*P* = .825). This discrepancy suggests that while smoking remains an independent predictor of mortality, its effect does not appear to be directly influenced by the AFII score. Adjusted Ferritin Inflammation Index, a composite score adjusting for inflammation, offers a broader and more comprehensive measure of mortality risk in HF patients, potentially reflecting multiple systemic factors beyond smoking. These findings further support the potential utility of AFII as a prognostic tool in HF management, providing more accurate risk stratification independent of specific risk factors such as smoking.

We also observed significant differences in survival rates based on AFII levels. Kaplan–Meier analyses revealed lower survival rates in patients with higher AFII levels across multiple subgroups, including NYHA class, outpatient/inpatient status, and overall. Notably, significant survival differences were found between patients with low and high AFII in both the NYHA Classes I and II and NYHA Classes III and IV groups. In the NYHA I and II group, higher AFII levels were associated with a more pronounced decline in survival. In the NYHA III and IV group, the survival rates for patients with high AFII were considerably lower compared with those with low AFII. Even in the NYHA Classes I and II patient group, where survival is expected to be relatively better, AFII levels could serve as an important marker in predicting the disease progression of these patients.

### Conclusion

Adjusted Ferritin Inflammation Index provides incremental prognostic information beyond conventional iron parameters by integrating markers of inflammation and nutrition. In our cohort, AFII, together with NYHA class, sodium, BNP, and smoking, independently predicted mortality, whereas ferritin and TSAT were not significant determinants of survival. These findings emphasize the limitations of traditional iron markers, particularly in the setting of chronic inflammation, and highlight AFII as a more accurate, inflammation-adjusted tool for risk stratification in patients with HFrEF and HFmrEF.

Although AFII was not used to guide therapy in this study, its consistent association with adverse outcomes suggests potential utility in identifying high-risk patients for closer monitoring and, with further validation, in informing iron repletion strategies. Prospective, multicentre validation is required to establish its clinical applicability and to determine its role as a complementary tool in guiding future therapeutic approaches.

### Limitations

Several limitations must be acknowledged. First, the retrospective, single-centre design restricts causal inference and limits generalizability to broader HF populations. Although our cohort included patients across different NYHA classes, the performance of AFII in HF with preserved ejection fraction or in inflammatory comorbid conditions remains untested. Second, while the sample size was sufficient for the primary analysis, subgroup comparisons (e.g. i.v. iron vs no i.v. iron) may have been underpowered.

Third, the AFII score was developed and validated within the same dataset. Despite internal validation via bootstrap and split-sample analysis, external validation in larger, independent cohorts is essential. Furthermore, although we assessed the association of i.v. iron therapy with mortality, it should be noted that treatment allocation was based on FAIR-HF criteria—not AFII—limiting causal interpretation.

Lastly, although we aimed to capture inflammation-adjusted iron status, other inflammatory biomarkers (e.g. IL-6, TNF-α, and hs-CRP) were not included due to lack of routine clinical availability. However, the use of widely accessible parameters (CRP, albumin, and ferritin) enhances AFII’s feasibility in real-world settings.

### Future directions

Future research should evaluate whether AFII-guided iron supplementation can better identify patients who benefit from therapy, particularly in cases with borderline ferritin or TSAT values. Incorporating additional inflammatory markers beyond CRP and albumin may enhance its predictive accuracy, and comparative studies with gold-standard methods, such as bone marrow biopsy, could further establish its clinical utility. Most importantly, prospective multicentre studies with external validation are essential to confirm the generalizability and clinical applicability of AFII before it can be adopted in routine practice.

## Supplementary Material

xvag028_Supplementary_Data

## Declarations

### Data Availability

The datasets generated during and/or analysed during the current study are available from the corresponding author on reasonable request. The authors assure that this paper has not been published before nor has been submitted for publication to another scientific journal.
