# Supplementary figures and images for: The adjusted ferritin inflammation index: a novel metric for predicting mortality in heart failure with reduced and mildly reduced ejection fraction
Source: ESC Heart Fail. 2026 Jan 21;13(1):xvag028. doi: 10.1093/eschf/xvag028 (PMC13108301; doi:10.1093/eschf/xvag028)

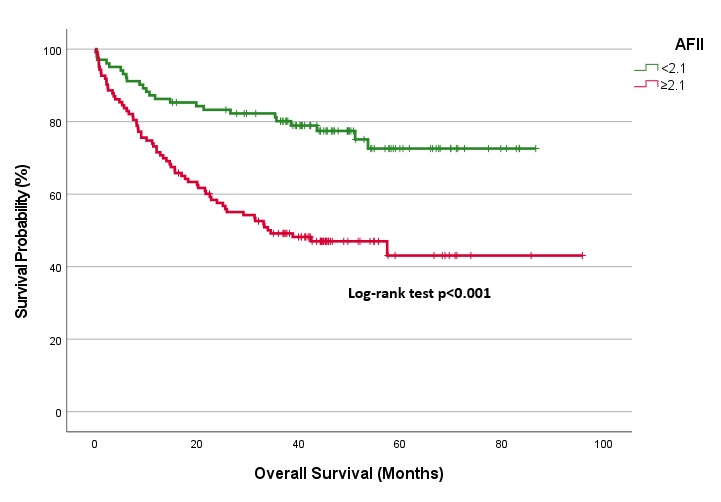

Supplement: xvag028_Supplementary_Data [file xvag028_supplementary_data.zip › Supplemantary Figure 1.jpg]

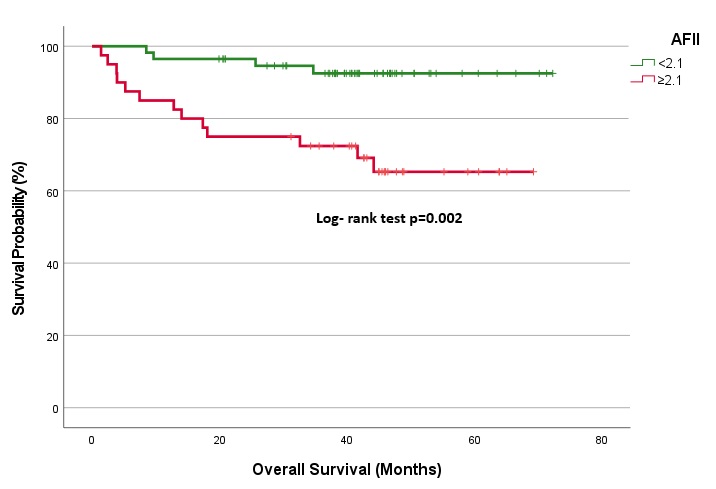

Supplement: xvag028_Supplementary_Data [file xvag028_supplementary_data.zip › Supplemantary Figure 2.jpg]
